# Comparative mineral and biochemical characterization of *Citrus reticulata* fruits and leaves to citrus canker pathogens, *Xanthomonas axonopodis*

**DOI:** 10.1186/s12870-024-05075-8

**Published:** 2024-05-09

**Authors:** Rab Nawaz, Abdul Ghani, Muhammad Nadeem, Toqeer Abbas, Anis Ali Shah, Shifa Shaffique, Hosam O. Elansary, Ihab Mohamed Moussa

**Affiliations:** 1 Department of Botany, University of Sargodha, Sargodha, Pakistan; 2 Institute of Food Science &Nutrition, University of Sargodha, Sargodha, Pakistan; 3 Department of Botany, Division of Science and Technology, University of Education, Lahore, Punjab Pakistan; 4 College of Agriculture & Life Science, School of Applied Biosciences, Kyungpook National University, 80 Daehak-ro, Buk-Gu, Daegu, 41566 Korea; 5 Plant Production Department, College of Food & Agriculture Sciences, King Saud University, Riyadh, 11451 Saudi Arabia; 6 Botany and Microbiology Department, College of Science, King Saud University, Riyadh, 11451 Saudi Arabia

**Keywords:** Citrus canker, Calcium, Minerals, Phosphorus, Chlorophyll, Sargodha, *Xanthomonos Axonopodis*

## Abstract

Pakistan’s economy greatly benefits from citrus production since these fruits are sold and consumed all over the world. Although citrus fruits are easy to cultivate, they are susceptible to diseases caused by bacteria, viruses, and fungi. These challenges, as well as difficulties in obtaining the proper nutrients, might negatively impact fruit yields and quality. Citrus canker is another complicated problem caused by the germ *Xanthomonas axonopodis*. This germ affects many types of citrus fruits all over the world. This study looked closely at how citrus canker affects the leaves and the quality of the fruit in places like Sargodha, Bhalwal, Kotmomin, and Silanwali, which are big areas for growing citrus in the Sargodha district. What we found was that plants without the disease had more chlorophyll in their leaves compared to the sick plants. Also, the healthy plants had better amounts of important minerals like calcium, magnesium, potassium, and phosphorus in their fruits. But the fruits with the disease had too much sodium, and the iron levels were a bit different. The fruits with the disease also didn’t have as much of something that protects them called antioxidants, which made them more likely to get sick. This study helps us understand how citrus canker affects plants and fruit, so we can think of ways to deal with it.

**Supplementary Information:**

The online version contains supplementary material available at 10.1186/s12870-024-05075-8.

## Introduction

Citrus species are fruit trees that have a significant economic impact on the world and have been tamed and grown for many years [1], and are distributed in worldwide in temperate and tropical regions, mainly on the continents of Southern Africa and Australia. A total of 1,943.7 thousand t of citrus fruits are produced in Pakistan over an area of 183.8 thousand hectares, with 94,806 t exported (Kinnow and others) to various countries [2].

Citrus fruit production is limited by a number of variables, including the prevalence of diseases brought on by bacterial, viral, fungal, and nematode pathogens. Citrus nematode, citrus gummosis, citrus canker, citrus greening, and citrus tristeza virus are among the prevalent diseases that harm citrus fruits in Pakistan. Citrus canker is one of these that is common and has a negative effect on plant growth and fruit quality in all citrus-growing regions of the world [3]. The causative agent of this disease is *Xanthomonas axonopodis* [4]. Although reports of citrus canker were first made in Punjab, Pakistan, the disease has now spread across the entire country. Unfortunately, all cultivars, including grapefruit, sweet oranges, lemons, limes, rootstocks, and their hybrids, are susceptible to this disease [5].

The canker-causing gram-negative bacterium only has one polar flagellum. It can withstand temperatures as high as 35–39 °C and grows aerobically in the 28–30 °C temperature range [6]. The surge in canker incidences in Brazil and other countries has been linked to insect-induced injuries that create new entry points for the pathogen, leading to merging lesions [5, 7]. The bacteria can also enter plants through stomata or wounds, including those caused by leaf miners on citrus leaves. Once within the plant, they proliferate and manifest as observable symptoms called necrotic patches. These lesion areas serve as a potent source of infection, facilitating further bacterial growth and increasing the risk of disease outbreaks in other parts of the plant or nearby plants [8]. This highlights how crucial it is to manage insect populations and stop lesions in order to successfully stop the spread of citrus canker.

Citrus canker manifests in various symptoms, such as pustules and necrotic lesions characterized by erupting corky tissues, surrounded by oily or water-soaked margins, and accompanied by a yellow halo. The severity of the disease leads to defoliation, dieback, premature fruit drop, and the development of blemished fruit [9]. Fruit lesions are especially important commercially since they make the fruit unfit for the fresh market, which drives down prices significantly. Developing disease-free stock, using copper sprays, and reducing pathogen spread through leaf miners or wind are examples of integrated control strategies for citrus canker [7, 10].

Citrus canker is known for its ease of transmission and its ability to spread rapidly. The primary mode of transmission is through water, especially wind-driven rain, which carries bacterial cells from infected plants to neighbouring healthy ones [11]. In addition, human action like handling or pruning contaminated plant material can spread the pathogens, as can contaminate tools, equipment and irrigation water [12]. The global movement of citrus plants and fruit has played a significant role in the spread of citrus canker to new regions, highlighting the importance of strict biosecurity measures and quarantines to prevent its introduction into disease-free areas [13]. Physiological characteristics of various citrus plants were analyzed, and results showed that citrus plants contain more chlorophyll as melatonin concentration increases [14].

Citrus canker is a formidable bacterial disease that poses a significant threat to Pakistan’s citrus plantations, causing economic losses due to reduced fruit quality and quantity. Despite on-going global efforts to control the disease, there is limited comprehensive literature on its impact on various citrus attributes. The effects of citrus canker on the morpho-physiological and biochemical characteristics of kinnow mandarin were thoroughly examined in this study. The study’s findings will offer valuable insights for urgent disease management strategies to safeguard the citrus industry and ensure food security.

## Materials and methods

### Study area description

Sargodha is known as the “California of Pakistan” for quality citrus production. The city has a harsh climate with scorching summers and freezing winters. The economy of Sargodha is based on agriculture and various industries. Four tehsils were selected for the samples collection and at each tehsil five different sub sites were explored.

### Sampling tehsils

A systematic approach was used to collect samples in District Sargodha’s four Tehsils: Silanwali, Bhalwaal, Kotmomin, and Sargodha. Each Tehsil provided five designated sub sites (Site A to E) for a total of 20 locations. Within each site, ten Kinnow plants aged 10 to 15 yrs were selected, half healthy; half infected; marked for subsequent sampling. Each plant yielded samples of twenty leaves and twenty fruits, serving as an individual replication. Harvesting occurred at commercial maturity, enabling a comprehensive assessment of plant attributes.

### Total antioxidant capacity (µmol /100 g) determination

A 0.1 ml sample solution containing a reducing species (in water, methanol, ethanol, dimethyl sulfoxide, or hexane) was combined with 1 ml reagent solution in an Eppendorf tube (0.6 M sulfuric acid, 28 mM sodium phosphate, and 4 mM ammonium molyb-date). The tubes were sealed and placed in a thermal block for ninety minutes at 95 °C. The samples’ absorbance was measured at 695 nm against a blank after they had cooled to room temperature. A standard blank solution was prepared as follows: 1 ml of reagent solution with an appropriate volume of a solvent that was similar to the one used for the sample. It was then incubated in the same manner as the other samples. For materials with unknown composition, lipid-soluble and water-soluble antioxidant abilities were determined using the a-tocopherol and ascorbic acid equivalents.

The acid-base digestion technique was used to calculate the amount of crude fibers [15]. Three grams of the oven-dried sample were taken, the fat was removed from the Soxhlet apparatus, and the sample was digested in distinct solutions of 1.25% H_2_SO_4_ and NaOH. Following filtration, the material underwent three rounds of washing with distilled water. The leftover material was then transferred to a china dish, and the crude fibers were measured after the dish was baked for 24 h at 105 °C. The amount of crude fibers in the sample explained the deviation in weights.

$$\text{C}\text{r}\text{u}\text{d}\text{e} \text{f}\text{i}\text{b}\text{e}\text{r} \left(\text{\%}\right)=\frac{\text{W}\text{e}\text{i}\text{g}\text{h}\text{t} \text{o}\text{f} \text{o}\text{v}\text{e}\text{n} \text{d}\text{r}\text{i}\text{e}\text{d} \text{s}\text{a}\text{m}\text{p}\text{l}\text{e}}{\text{W}\text{e}\text{i}\text{g}\text{h}\text{t} \text{o}\text{f} \text{f}\text{r}\text{e}\text{s}\text{h} \text{s}\text{a}\text{m}\text{p}\text{l}\text{e}}\times 100$$

### Mineral Assessment (mg/kg)

After being baked, the fruit samples were ground into a fine powder and put through a wet digesting procedure. After adding 10 ml of HNO3 to each 0.5 g sample in a digestion flask, the samples were kept there for the entire night. Following that, 5 ml of perchloric acid were added to the sample and the digestion process was conducted on a hot plate. The procedure was repeated until the sample solution was clear. After that, 50 ml of the final solution; which was subsequently submitted for analysis, was created by adding up to 100 ml of distilled water. Then standard solutions were created, and the digested samples were ready for elemental analysis using those standards. The produced sample solution was fed into the atomic absorption spectrophotometer for elemental analysis. For each metal, a standard curve was created by running samples of known strength. The elemental composition of the samples was calculated using the standard curves created for each metal using the procedure [15]. Standard approaches were used to estimate Calcium (Ca), Magnesium (Mg), Phosphorus (P), Iron (Fe), Sodium (Na), and Potassium (K). The given formula was used to make 1000 ppm concentration stock solutions for each metal.

$$X =\frac{\text{M}\text{o}\text{l}\text{e}\text{c}\text{u}\text{l}\text{a}\text{r} \text{w}\text{e}\text{i}\text{g}\text{h}\text{t} \text{o}\text{f} \text{s}\text{a}\text{l}\text{t}}{\text{M}\text{o}\text{l}\text{e}\text{c}\text{u}\text{l}\text{a}\text{r} \text{w}\text{e}\text{i}\text{g}\text{h}\text{t} \text{o}\text{f} \text{m}\text{i}\text{n}\text{e}\text{r}\text{a}\text{l}}\times 0.1$$

For the production of the standard curve, X grams of salt were dissolved in 100 ml distilled H_2_O to produce a 1000 ppm solution, which was then diluted to a 100-ppm solution. A diluted solution of 1 ppm to 10 ppm was used to standardize the equipment.

### Statistical analysis

Utilizing SPSS 20, the data was submitted to a Two-way Analysis of Variance (Completely Randomized Design, 2 variables) in order to determine the significance of the differences between means and interactions. For significant factors, Tukey HSD all-pairwise comparisons were used to compare means at the 5% level of significance. Microsoft Excel 2010 was used to prepare the graphs.

## Results

### Assessment of physiological attributes in diseased and healthy plants

#### Estimating the impact of Citrus canker on biochemical properties of citrus

The biochemical properties of both healthy and diseased fruits were determined, and the results show that the highest level of CFC was found in the Bhalwal region for healthy fruits and the highest level in Silanwali for diseased fruits (Table 1). The results reveal a strong statistical significance in total antioxidant activity concerning the interplay between disease factors and the geographical administrative divisions known as “tehsils.” Furthermore, an evident interaction between tehsil-specific attributes and disease conditions has been established, showcasing a substantial impact on total antioxidant activity. For instance, at Bhalwal, TAA in healthy fruits was 1015.0 µmol/100 g, while in diseased fruits; it was 526.0 µmol/100 g. Fruits that were affected had TAA values that were around 50 times greater than those of healthy fruits. Tehsil analysis also revealed notable variations in TAA values at the tehsil level.

**Table 1:** Determination of biochemical property in healthy and diseased fruits

Parameters	Tehsil	Healthy	Diseased	Mean
TAA (µmol /100 g)	Bhalwal	1, 015.0 ± 35.27a	526.0 ± 29.56d	770.5 ± 84.34 C
	Kotmomin	1, 054.8 ± 55.67a	633.5 ± 40.00c	844.1 ± 77.30B
	Sargodha	1, 004.8 ± 23.05a	839.8 ± 20.61b	922.3 ± 31.12 A
	Silanwali	1, 087.8 ± 11.74a	435.7 ± 32.31d	761.7 ± 109.89 C
	Mean	1, 040.6 ± 17.92 A	608.7 ± 37.45B	
CFC	Bhalwal	6.95 ± 0.111	5.58 ± 0.122	6.27 ± 0.240 A
	Kotmomin	6.53 ± 0.120	5.60 ± 0.122	6.06 ± 0.175 A
	Sargodha	6.87 ± 0.214	5.51 ± 0.220	6.19 ± 0.269 A
	Silanwali	6.71 ± 0.154	5.74 ± 0.050	6.23 ± 0.178 A
	Mean	6.76 ± 0.080 A	5.61 ± 0.068B	

### Mineral contents comparison in bacterial affected and healthy fruits

There is a statistically significant association between calcium levels and disease conditions, as well as potassium and iron levels. In the context of interactions between tehsil attributes and disease conditions, calcium, iron, and potassium exhibit a remarkably robust statistical significance (*p* < 0.01). Delving into the interaction dynamics between tehsil aspects and disease conditions, calcium retains its significance; iron retains its high level of statistical significance, while potassium’s impact becomes statistically non-significant (Table 2).

**Table 2:** Evaluation of calcium, iron and potassium in healthy/diseased fruits

Parameters	Tehsil	Healthy	Diseased	Mean
**Calcium (mg/kg)**	Bhalwal	187.76 ± 8.36a	143.96 ± 2.10b	165.86 ± 8.36 A
	Kotmomin	135.00 ± 2.20b	105.80 ± 3.51d	120.40 ± 5.24B
	Sargodha	185.36 ± 5.90a	146.03 ± 2.69b	165.70 ± 7.23 A
	Silanwali	119.50 ± 2.83c	100.53 ± 1.96d	110.01 ± 3.55 C
	Mean	156.91 ± 7.36 A	124.08 ± 4.97B	
**Iron (mg/kg)**	Bhalwal	1.12 ± 0.068ef	0.90 ± 0.076f	1.01 ± 0.060 C
	Kotmomin	1.60 ± 0.047d	1.28 ± 0.057e	1.44 ± 0.064B
	Sargodha	2.72 ± 0.093a	1.86 ± 0.095c	2.29 ± 0.157 A
	Silanwali	2.57 ± 0.109a	2.21 ± 0.089b	2.39 ± 0.090 A
	Mean	2.00 ± 0.158 A	1.56 ± 0.122B	
**Potassium (mg/kg)**	Bhalwal	2, 011.34 ± 57.98	1, 944.86 ± 53.31	1, 978.10 ± 38.75 A
	Kotmomin	1, 663.34 ± 55.06	1, 601.33 ± 53.81	1, 632.34 ± 37.74 C
	Sargodha	1, 835.34 ± 30.54	1, 730.11 ± 52.69	1, 782.72 ± 33.64B
	Silanwali	1, 362.54 ± 46.22	1, 286.16 ± 67.29	1, 324.35 ± 40.53D
	Mean	1, 718.14 ± 59.27 A	1, 640.61 ± 60.69B	

NS = (*P* > 0.05)

**Fig. 1:**
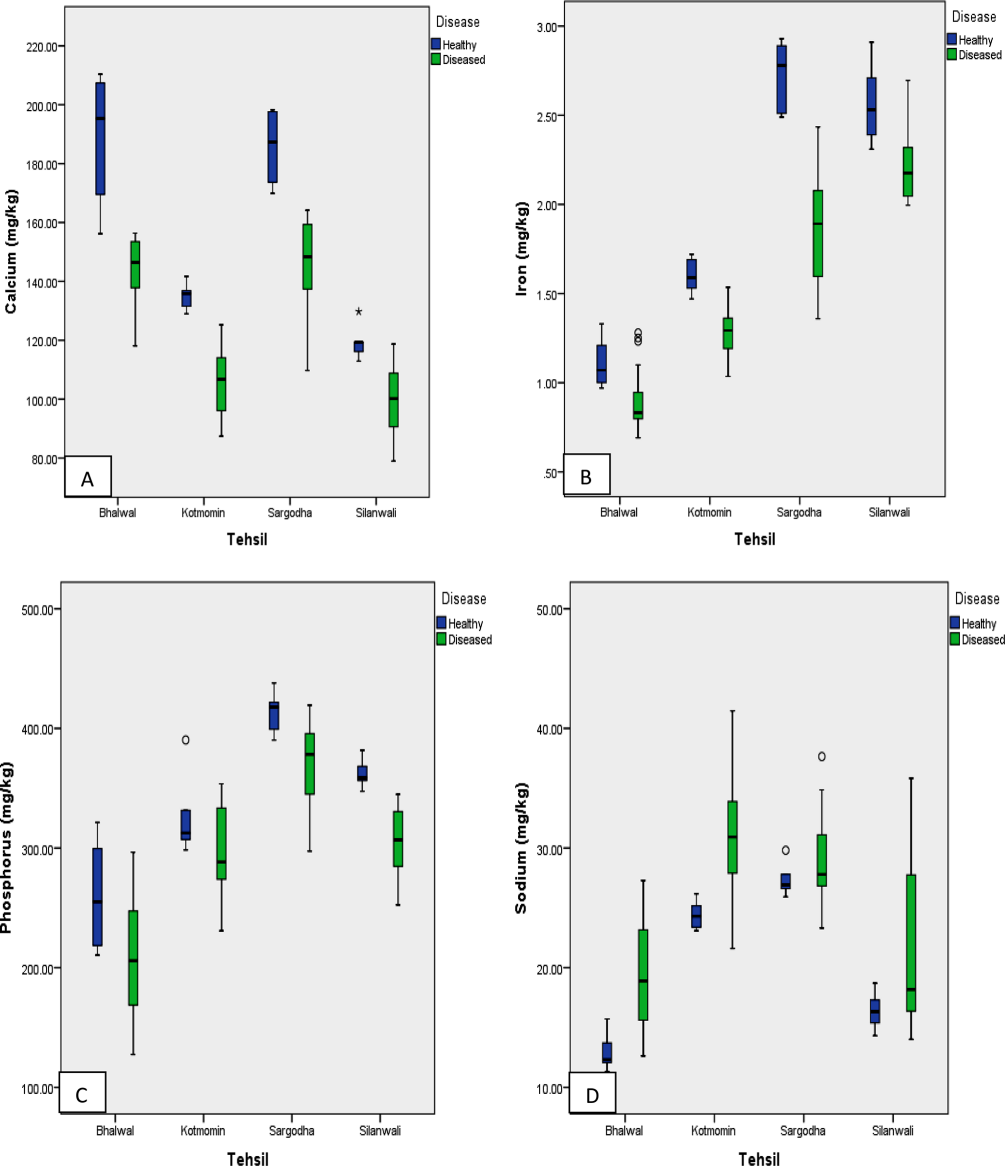
(**A**-**D**): Box plot for Ca, Fe, P, and Na conc. in healthy and diseased fruits of studied tehsils

Results illustrates that calcium concentration was the highest in healthy fruits compared to diseased fruits across all tehsils, with significant differences between the two conditions (Fig. 1A). Furthermore, for both healthy and diseased fruits, the highest calcium levels were found in Tehsil Bhalwal, while the lowest levels were found in Tehsil Silanwali. Tehsils Kotmomin and Silanwali showed the least fluctuation in calcium concentration among healthy fruits, unlike other tehsils.

Results confirmed Bhalwal as the site where the highest calcium value was documented for healthy fruits and Silanwali as the site with the lowest, both for healthy and diseased fruits. On the other hand tehsil Sargodha had the highest calcium value for diseased fruits. The mean value of calcium in healthy fruits significantly differed from the accumulative mean of diseased fruits, with tehsils Bhalwal and Sargodha having the highest mean values, and tehsil Silanwali having the lowest mean value (Table 2).

Graphical data depicted that iron concentration was the highest in healthy fruits compared to diseased fruits across all tehsils, showing a substantial difference between the two conditions. The highest iron concentration was measured in Tehsil Sargodha, while the lowest iron concentration was found in Tehsil Bhalwal (Fig. 1B). Phosphorus concentration was higher in healthy fruits compared to infectious fruits in all tehsils, with a slight difference between the two. Tehsil Sargodha displayed the maximum phosphorus value, while tehsil Bhalwal displayed the lowest, showing fluctuations mainly in tehsil Bhalwal (Fig. 1C), while the Sodium concentration was high at Kotmomin Tehsil in healthy fruits (Fig. 1D). Tehsil Sargodha had the highest iron concentration for healthy fruits, according to the results, whereas Tehsil Silanwali had the highest concentration for unhealthy fruits. On the other hand, the lowest iron concentration was found in tehsil Bhalwal in both healthy and diseased fruits (Table 2).

**Fig. 2:**
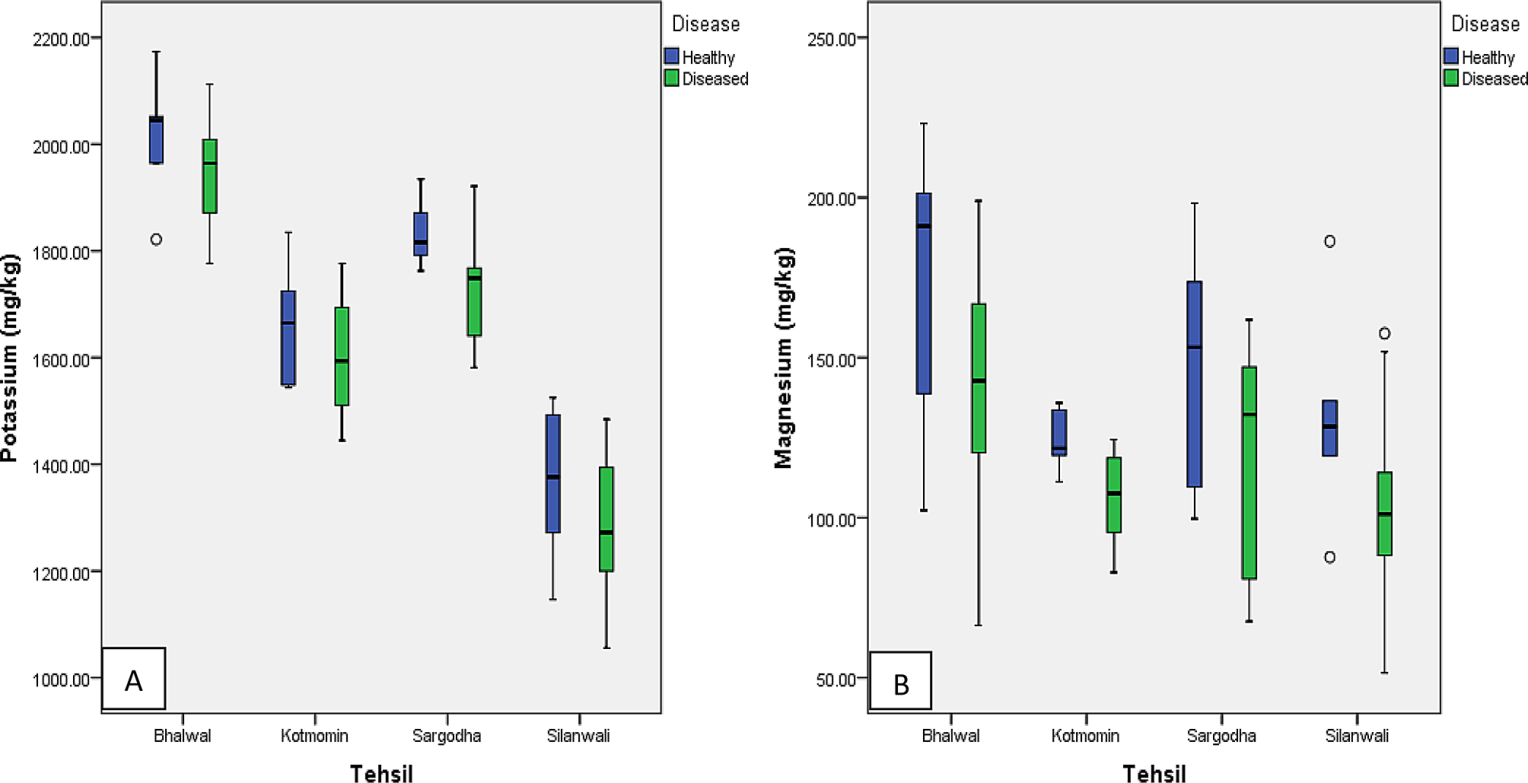
(**A**-**D**): Box Plot for K (**A**) and Mg (**B**) conc. in healthy and diseased fruits of studied tehsils

Significant differences in potassium levels between tehsils were displayed graphically. In tehsils Sargodha and Kotmomin, both healthy and diseased fruits had moderate potassium levels; tehsil Bhalwal had the highest amounts, and tehsil Silanwali had the lowest. Overall, potassium concentration was slightly higher in healthy fruits compared to diseased fruits across all tehsils (Fig. 2A). The concentration of magnesium was slightly higher in healthy fruits compared to diseased fruits in all tehsils (Fig. 2B).

The potassium interaction table between diseased fruits and tehsils showed that the mean potassium value in healthy fruits was different from the mean value of all the tehsils for diseased fruits. The highest potassium concentration in both healthy and diseased fruits was present at tehsil Bhalwal and the lowest in tehsil Silanwali. Tehsil Bhalwal’s mean value had a significant difference from the other three tehsils (Table 2). Similarly, the interactions between tehsil-specific attributes and disease factors yield consistently high levels of statistical significance for magnesium, sodium, and phosphorus. However, focusing on the interplay between tehsil characteristics and disease conditions, sodium remains highly significant, whereas phosphorus and magnesium show statistical non-significance interaction (Table 3).

**Table 3:** Evaluation of magnesium, sodium and phosphorus in healthy/diseased fruits

Parameters	Tehsil	Healthy	Diseased	Mean
**Magnesium (mg/kg)**	Bhalwal	171.28 ± 16.97	139.10 ± 6.17	155.19 ± 10.06 A
	Kotmomin	124.37 ± 4.58	106.12 ± 4.79	115.25 ± 4.36B
	Sargodha	146.89 ± 10.39	115.92 ± 6.39	131.40 ± 7.73B
	Silanwali	131.65 ± 4.56	102.15 ± 3.19	116.90 ± 5.57B
	Mean	143.55 ± 6.33 A	115.82 ± 4.09B	
**Sodium (mg/kg)**	Bhalwal	13.03 ± 0.35 g	18.93 ± 0.54e	15.98 ± 1.03 C
	Kotmomin	24.42 ± 0.57c	31.17 ± 0.92a	27.80 ± 1.24 A
	Sargodha	27.42 ± 0.67b	28.77 ± 0.64b	28.09 ± 0.49 A
	Silanwali	16.41 ± 0.76f	21.40 ± 0.67d	18.90 ± 0.96B
	Mean	20.32 ± 1.36B	25.07 ± 1.20 A	
**Phosphorus (mg/kg)**	Bhalwal	261.02 ± 11.31	211.40 ± 04.28	236.21 ± 10.04 C
	Kotmomin	328.00 ± 16.50	295.39 ± 18.13	311.70 ± 12.77B
	Sargodha	413.37 ± 08.45	372.49 ± 15.83	392.93 ± 10.86 A
	Silanwali	362.52 ± 05.84	305.54 ± 09.38	334.03 ± 10.83B
	Mean	341.23 ± 13.71 A	296.20 ± 14.44B	

NS = (*P* > 0.05)

The results of the analysis of variance (mean squares) for the TAA and crude Fiber content indicate that the interaction between the two variables was extremely significant (Table 4). Furthermore, it was found that tehsil Bhalwal exhibited the highest magnesium concentration for both healthy and diseased fruits, while tehsil Kotmomin demonstrated the lowest concentration for healthy fruits. Tehsil Silanwali had the lowest magnesium concentration, comparable to healthy fruits, whereas Tehsil Bhalwal had the highest magnesium concentration in relation to diseased fruits. It was discovered that the mean magnesium value of all the tehsils for unhealthy fruits was much different from the mean value of magnesium in healthy fruits. The mean value for both healthy and diseased fruits was highest in tehsil Bhalwal out of all tehsils, while it was lowest in tehsil Kotmomin. The mean value for Tehsil Bhalwal differed significantly from that of all other tehsils (Table 5).

**Table 4:** Analysis of variance (mean squares) table for crude fiber content and TAA

Source of variation	Degrees of freedom	Mean squares	
		Crude fiber content	TAA
Disease	1	13.3749**	1865087.0**
Tehsil	3	0.0763^NS^	56025.0**
Disease*Tehsil	3	0.1416^NS^	102595.0**
Error	32	0.1105	5596
Total	39		

NS = Non-significant (*P* > 0.05); * = Significant (*P* < 0.05); ** = Highly significant (*P* < 0.01)

**Table 5:** Analysis of variance (mean squares) table regarding evaluated minerals

Source of variation	Degrees of freedom	Mean squares		
		Calcium (mg/kg)	Iron (mg/kg)	Potassium (mg/kg)
Disease	1	10774.5**	1.94437**	60100.0*
Tehsil	3	8704.7**	4.46710**	760571.0**
Disease*Tehsil	3	306.7*	0.20813**	943
Error	32	90.9	0.03339	14,064
Total	39			
**Source of variation**	**Degrees of freedom**	**Mean squares**		
		**Magnesium (mg/kg)**	**Sodium (mg/kg)**	**Phosphorus (mg/kg)**
Disease	1	7684.26**	225.492**	20270.3**
Tehsil	3	3418.10**	382.304**	41993.2**
Disease*Tehsil	3	102.83^NS^	14.084**	279.3^NS^
Error	32	343.72	2.182	744.4
Total	39			

NS = Non-significant (*P* > 0.05); * = Significant (*P* < 0.05); ** = Highly significant (*P* < 0.01)

**Fig. 3:**
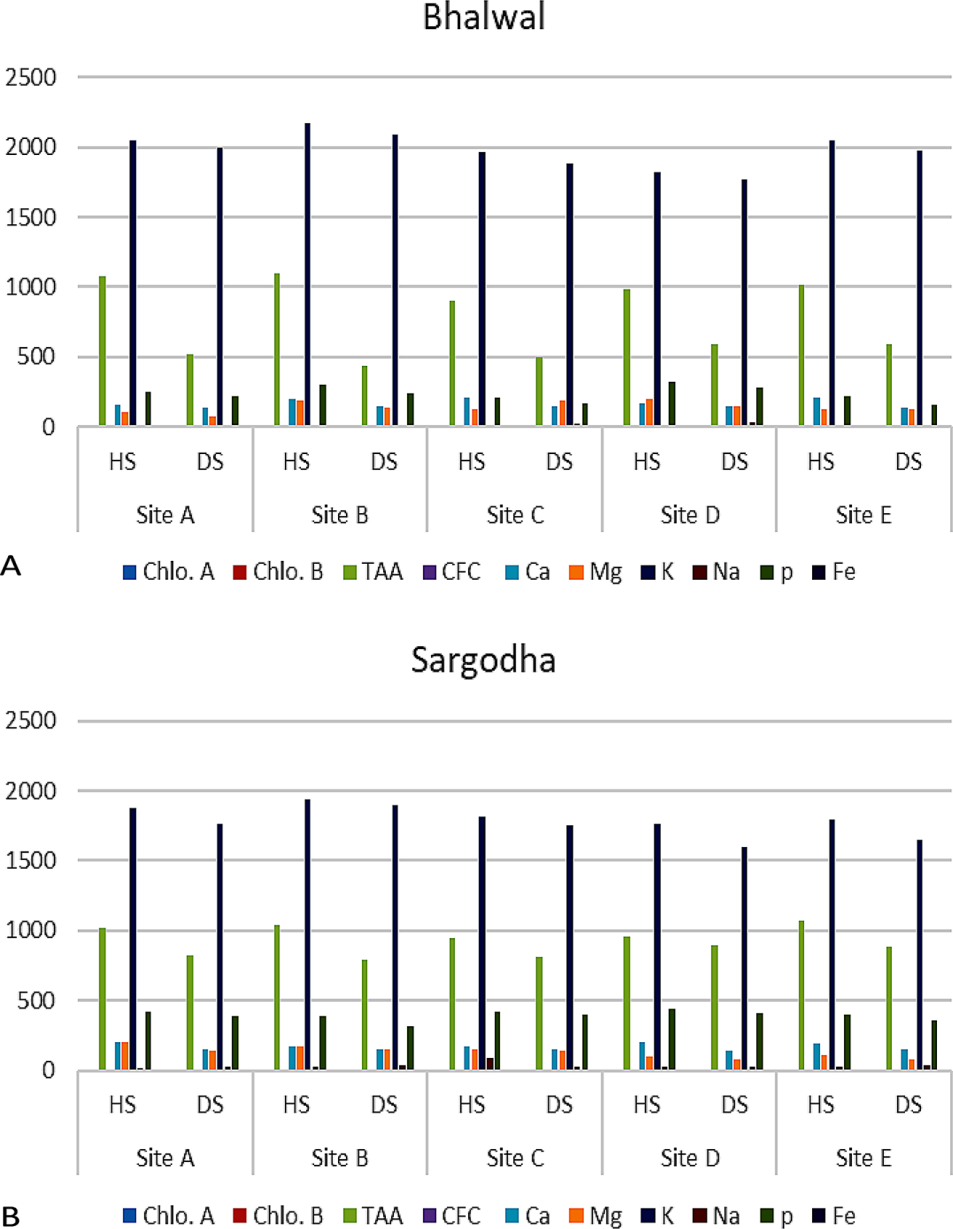
(**A**-**B**): Comparisons of evaluated attributes at various sites of T_1_ Bhalwal (**A**) and T_2_ Sargodha (**B**), HS-healthy samples, DS-disease samples, Chlo.A-Chlorophyll-a, Chlo.B- Chlorophyll-b, TAA-Total Amino Acids, CFC-Crude Fiber Contents, K-Potassium, Na-Sodium, P- Phosphorus, Mg-Magnesium, Ca-Calcium,) (T_1_- Tehsil Bhalwal, T_2_- Tehsil Sargodha)

Across all tehsils, graphical data shows that the concentration of sodium was higher in diseased fruits than in healthy fruits. Tehsil Silanwali was linked to the lowest sodium content, and Tehsil Kotmomin was linked to the highest. Fluctuations were prominent in sodium concentration in diseased fruits of tehsil Silanwali (Fig. 3A). In the context of mineral composition, elements such as calcium (Ca), magnesium (Mg), phosphorus (P), potassium (K), and iron (Fe) exhibited elevated concentrations across all healthy samples from the studied sites (Fig. 3B).

**Fig. 4:**
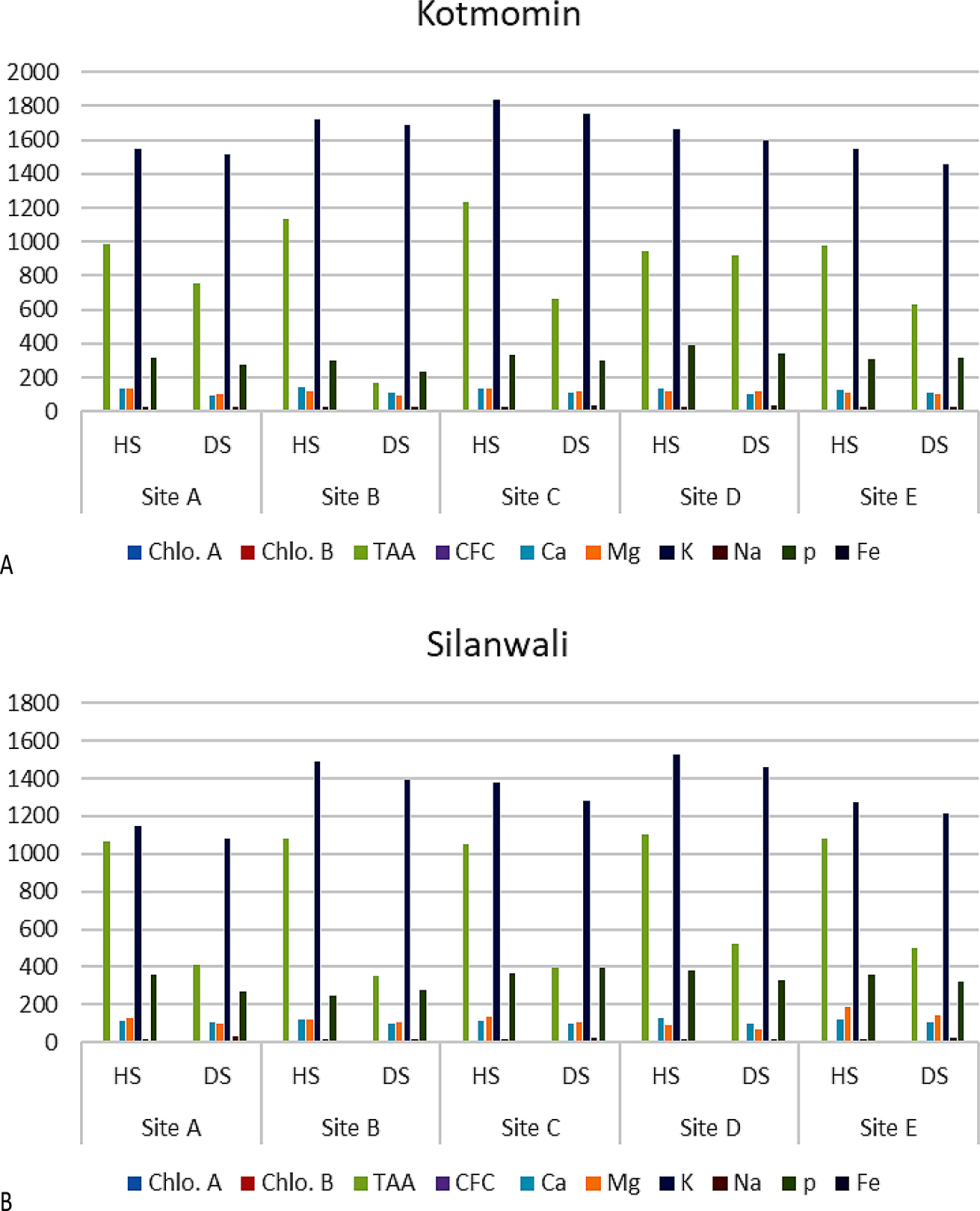
(**A**-**D**): Comparisons of evaluated attributes at various sites of T_3_ Kotmomin (**A**) and T_4_ Silanwali (**B**). HS-healthy samples, DS- disease samples, Chlo.A-Chlorophyll-a, Chlo.B-Chlorophyll-b, TAA-Total Amino Acids, CFC-Crude Fiber Contents, K-Potassium, Na-Sodium, P- Phosphorus, Mg-Magnesium, Ca-Calcium)

**Fig. 5:**
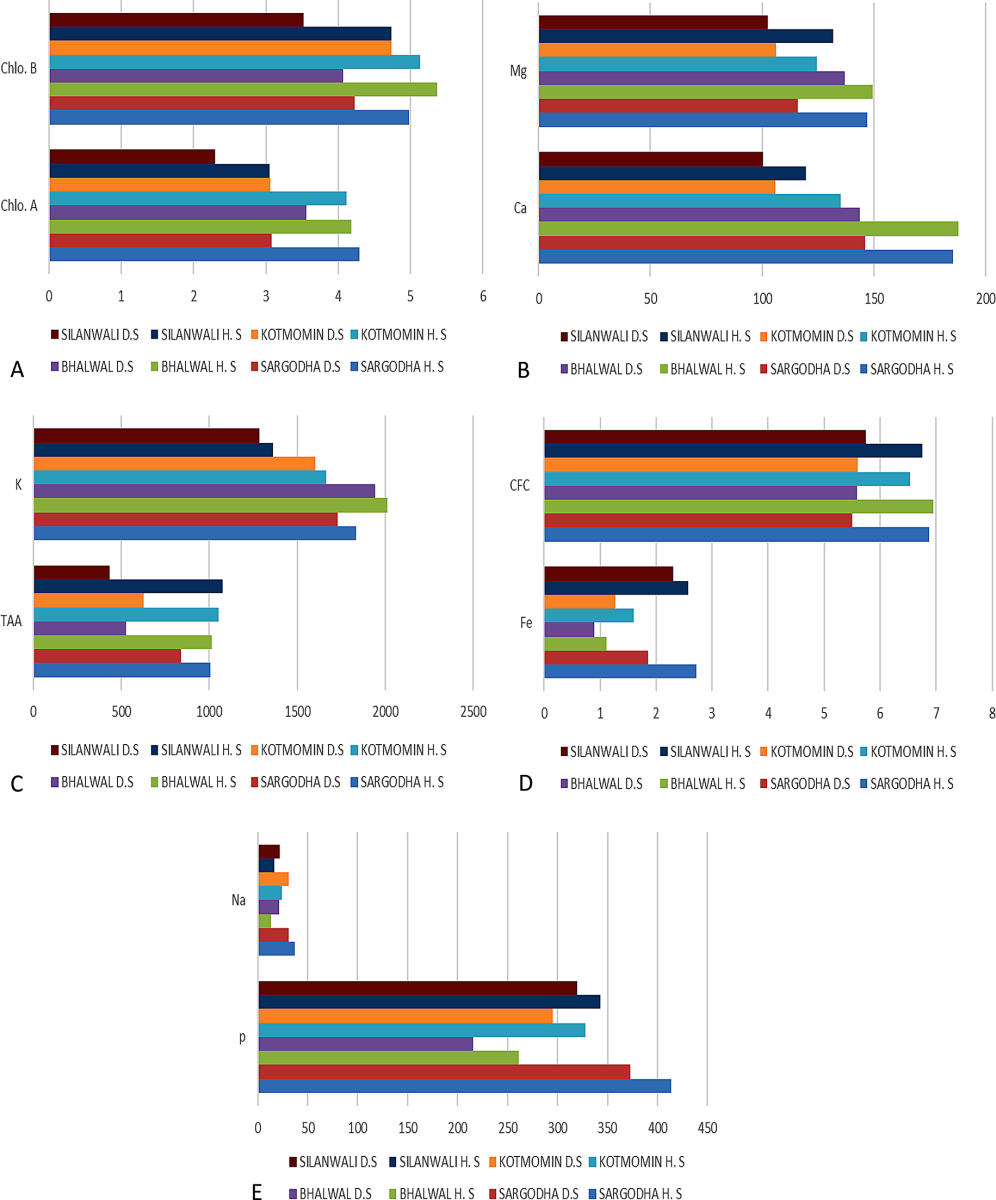
(**A**-**E**): Comparisons of tehsils with respect to photosynthetic pigments, minerals, CFC and total antioxidant activity. HS-healthy samples, DS- disease samples, Chlo.A-Chlorophyll-a, Chlo.B- Chlorophyll-b TAA-Total Amino Acids, CFC-Crude Fiber Contents, K-Potassium, Na-Sodium, P- Phosphorus, Mg-Magnesium, Ca-Calcium)

Across the investigated sites, it was shown that samples classified as diseased had higher quantities of sodium (Na), indicating a discernible difference. Furthermore, a consistent trend resembling the aforementioned observations was also noted within the studied sites of tehsil Silanwali and tehsil Sargodha (Fig. 4A and B). This conformity in results further supports the validity and significance of the identified patterns across multiple locations of district Sargodha. In summary, the results indicate a strong association between the health status of the samples and the recorded parameters, with higher levels of TAA, CFC, and select mineral compositions being linked to healthy samples. This information provides valuable insights into the potential factors influencing the health of the studied sites within the aforementioned regions (Fig. 5A and E).

### Comparison of sub-sites in each studied tehsil with respect to Chlorophyll a and b, na, Fe and crude fiber content

**Fig. 6:**
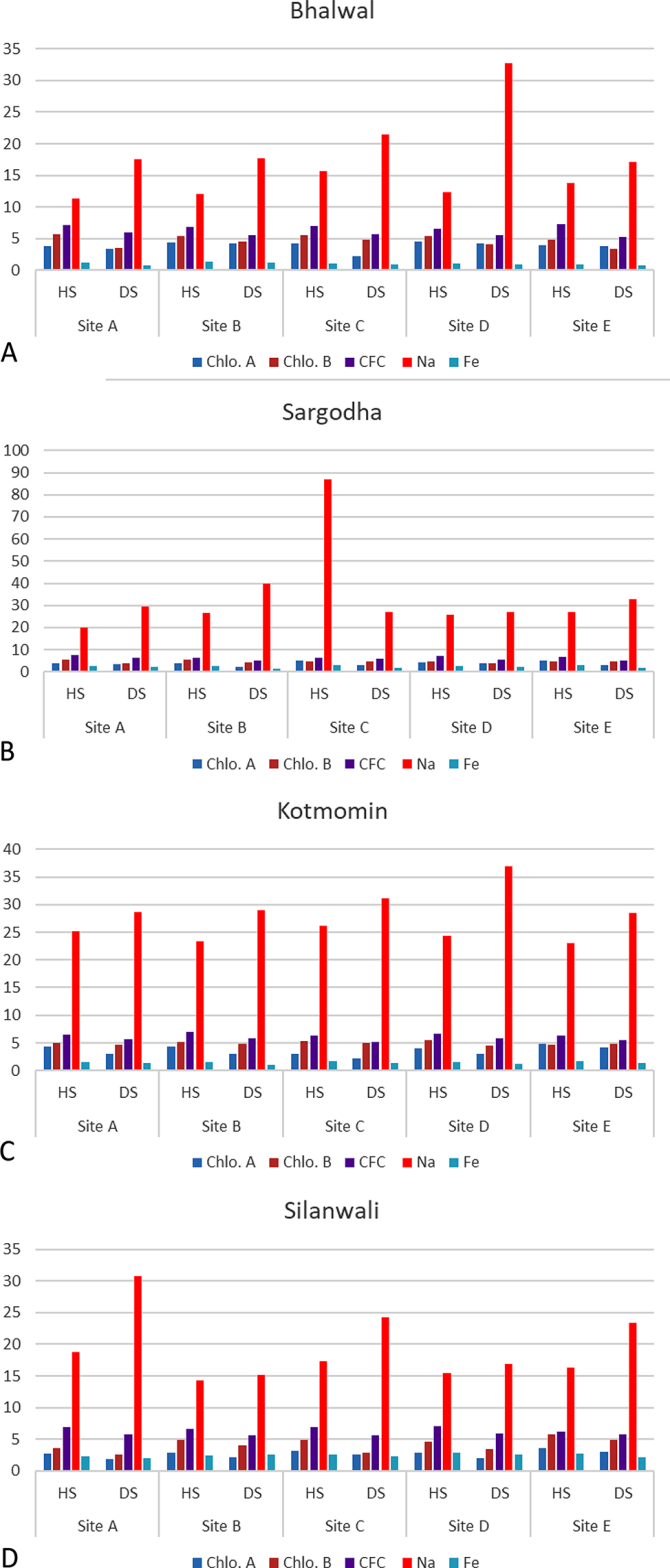
Comparison of sites in each studied tehsil with respect to photosynthetic pigments, minerals and crude fiber content. HS-healthy samples, DS- disease samples, Chlo.A-Chlorophyll-a, Chlo.B-Chlorophyll-b TAA-Total Amino Acids, CFC-Crude Fiber Contents, K-Potassium, Na-Sodium, P- Phosphorus, Mg-Magnesium, Ca-Calcium).

Graphical data for the sub-sites at different tehsils show that the highest concentrations of sodium as present at sub-site D at Bhalwal tehsil in diseased fruits while the highest concentration of chlorophyll b was recorded at sub-site A at Bhalwal tehsil in healthy fruits (Fig. 6A). The highest concentration of sodium as present at sub-site C at Sargodha tehsil in healthy fruits while the highest concentration of CFC was recorded at sub-site A at Sargodha tehsil (Fig. 6B). The highest concentration of sodium as present at sub-site D at Kotmomin tehsil in diseased fruits while the highest concentration of CFC was recorded at sub-site A at Kotmomin tehsil (Fig. 6C). The highest contents of sodium were recorded at sub-site A at Silanwali tehsil in diseased fruits while the highest concentration of CFC was recorded at sub-site C at Silanwali tehsil (Fig. 6D).

## Discussion

Citrus canker is a major issue in citrus cultivation because it damages leaves, fruits, and twigs and lowers the general health of the plants. Our research aligns with Stall et al.‘s findings, showing that citrus canker impacts multiple parameters [16]. Our research clearly shows that citrus canker lowers fruit harvests by decreasing tree vigour and productivity. Fruits from canker-affected trees may experience up to a 30% decrease in fresh market pack out rates.

### Photosynthetic pigments assessment

Chlorophyll a and b levels in *Citrus reticulata* leaves from different tehsils in Sargodha district were higher in healthy leaves compared to Xanthomonas infected leaves, attributed to citrus canker infection. Variations in chlorophyll contents among healthy leaves across tehsils may be influenced by farmed regions, growth conditions, soil type, seasonal fluctuations, cultivars, and storage conditions. Bacterial infected leaves exhibited significantly reduced chlorophyll concentration compared to healthy leaves. Citrus canker-affected leaves and branches also showed signs of yellowing and chlorosis. Previous studies also reported a substantial decrease in chlorophyll concentration due to citrus canker infection and viral illnesses like *Chilli veinal* mottle virus [17]. Reduced chlorophyll content leading to chlorosis is a common sign in various plant diseases.

Distinct variation in the concentration of chlorophyll between citrus canker-infected and healthy leaves, which have been caused by bacterial infections and leaf miner attacks, was noticed. In a study on citrus it was also found a link between short-duration, high-intensity rainstorms and disease increase. The decreased chlorophyll a concentration in *Citrus reticulata* leaves (0.59 mg/g), differing from our observations for non-diseased leaves, which may be due to genetic variability, leaf age, photoperiod, and soil texture [14, 18, 19].

Chlorophyll a content was measured in NML and ML. In Sargodha, TTS, and Vehari, NML showed 1.56, 1.75, and 1.33 mg/g FW, while ML exhibited 1.04, 1.07, and 0.8 mg/g FW, respectively [20]. In Tehsil Silanwali, Kotmomin, Bhalwal, and Sargodha, NML recorded 3.05, 4.11, 4.18, and 4.29 mg/g, and ML showed 2.30, 3.07, 3.56, and 3.09 mg/g, respectively. The decreased chlorophyll-a concentration was measured in diseased leaves due to leaf miner attack and CC infections. The values in our study were higher, possibly due to genetic diversity, leaf age, foliar micronutrient administration (Zn, Cu, and B), photoperiod, or soil texture [20].

Our findings were slightly higher than those of [21], possibly due to an increased ratio between chlorophyll a and b with higher light intensity. Shading did not affect the anatomical characteristics of Ponkan mandarin plants [22], but chlorophyll levels differed in sun and shade leaves.

### Assessment of antioxidant activity and crude fiber content

DPPH (1, 1-diphenyle-1-2-picrylhydrazyl) inhibition was used to assess antioxidant activity in relation to seasonal fluctuations (spring, summer, and autumn flushes). This assessment was performed on two distinct groups: NML (with inhibition percentages of 27.66%, 32.27%, and 45.23% for the respective flushes) and ML (with inhibition percentages of 18.12%, 18.07%, and 32.40% for the corresponding flushes), as detailed in the work by [20]. Investigations on diseased and non-diseased fruits revealed a consistent pattern, with healthy fruits showing higher antioxidant activity than their diseased counterparts [20]. This disparity can be attributed to potential infections and disease manifestations. The primary factor influencing the reduced antioxidant activity in diseased fruits appears to be associated with the onset of citrus canker (CC) infection. This phenomenon can be attributed to the degradation of chlorophyll in CC-affected plants, leading to a decline in photosynthetic activity. This deterioration of antioxidant activity could potentially be attributed to the oxidative deprivation of vital bioactive components such as vitamin C, carotenoids, and phenolic compounds.

The flavedo area of *Citrus sinensis* had a significant amount of crude fiber (13.43% ± 0.03%) [23]. These results suggest that the differences in crude fiber content between healthy and ill apples are not as great as previously thought. These variations may be explained by variations in the nutritional makeup of the nutrients. Furthermore, our research reveals slight variations in crude fiber content between diseased and non-diseased fruits, with the former exhibiting relatively lower values. These observed distinctions between leaves of diseased and non-diseased plants may be attributed to factors such as leaf miner infestations and CC infections.

### Determination of minerals

The elemental profile of *Citrus reticulata* from four tehsils in District Sargodha demonstrated the significance of potassium (K) for photosynthesis, protein synthesis, and the synthesis of carbohydrates, fruiting, and the general health of plants. A deficiency in K can slow down photosynthesis and lead to disease problems, while an imbalance with nitrogen (N) can cause protein build-up and fruit-related issues. Potassium also plays a vital role in various bodily processes, acting as an electrolyte for cardiac function, digestion, and muscular contraction [24, 25].

The study’s observations on potassium concentration in *Citrus reticulata* fruits differ from the values reported by [26], where our findings were lower for both healthy and diseased fruits. Citrus nutritional content can vary due to factors such as farmed regions, soil type, cultivars, and storage conditions [27]. Potassium concentration was higher in healthy fruits compared to diseased fruits, possibly influenced by infection. Potassium plays a crucial role in various cell processes, affecting stomata function, photosynthesis, and plant turgor [28]. Fertilizer use and soil composition may be the cause of variations in potassium concentration between tehsils.

In plants, photosynthesis and chlorophyll depend on magnesium (Mg). It is difficult to identify magnesium shortage in the early stages of growth. There are differences between the results of our magnesium concentration test and earlier research [29]. The concentration of magnesium was measured in diseased fruits122 mg/kg [26]. Healthy fruits exhibited higher magnesium levels than diseased ones, potentially influenced by infections. Differences in the mineral composition of the soil could be the cause of variations in the concentration of magnesium in different tehsils [26, 29].

The study found the highest phosphorus concentration in citrus pulp, with a small amount also present in fruit skin. Our results related to phosphorus levels were higher than those reported by [30], who found 12 mg/100 g in oranges, 8 mg/100 g in grapefruit, and 16.2 mg/100 g in lemon. However, our findings differed from [26], who reported 181 mg/kg. The variation in phosphorus content in *Citrus reticulata* fruit could be attributed to soil composition and fertilizer application across different tehsils. Calcium levels were also higher in our study compared to [26], with higher concentrations in non-diseased fruits, possibly influenced by soil composition and fertilizer usage. Calcium contents were the highest in healthy fruits compared to diseased ones, and differences in calcium content among tehsils may be due to soil composition and genetic variability. Sodium concentrations showed discrepancies with [26], with lower values in healthy fruits and potential links to soil composition and fertilizer use. Overall, the variations observed may be influenced by infection [26, 30].

Iron is crucial for human health as it forms a part of hemoglobin, facilitating oxygen transport to body tissues. Citrus juices have relatively low iron content, but supplementation in drinks can aid iron deficiency. The iron concentrations were slightly higher in our results than [26], who reported 0.98 mg/kg. Variations in iron content may be attributed to soil composition, climate changes, and fertilizer usage. Overall, healthy fruits exhibited lower sodium contents than diseased ones, possibly influenced by infection. Differences in sodium content across tehsils may be related to soil mineral concentrations and fertilizer application [26].

## Conclusion

Citrus canker continues to be a significant challenge for citrus industries worldwide. Its rapid spread, destructive impact, and economic repercussions make it a constant concern for citrus growers, researchers, and policymakers alike. Implementing stringent biosecurity measures, conducting thorough research, and fostering international collaboration are essential to mitigating the threat of citrus canker and safeguarding the health and prosperity of the global citrus industry.

### Recommendations

To manage citrus canker effectively, enforce strict quarantine measures, regularly inspect and monitor orchards, remove infected trees, and promote disease-resistant citrus varieties. Implement proper sanitation practices and educate farmers and workers about canker management. Utilize copper-bactericides in high-risk areas, limit movement of infected material, and collaborate with agricultural authorities for an efficient management plan, while supporting sustainable research solutions for long-term control.

(HS-healthy samples, DS- disease samples) (T_3_- Tehsil Kotmomin, T_4_- Tehsil Silanwali).

### Electronic supplementary material

Below is the link to the electronic supplementary material.

Supplementary Material 1

## Data Availability

All data generated or analyzed during this study are included in this published article [and its supplementary information files].

## Declarations

### Voucher specimens

Citrus specimens were identified by Dr. Abdul Ghani associate professor of Botany, University of Sargodha, Sargodha, Pakistan and Dr. Mansoor Hameed, Professor at University of Agriculture Faisalabad. Herbarium sheets (number S2023/3421–3467) are deposited at Department of Botany University of Sargodha, Sargodha Pakistan.

## References

[1] Shi W, Song W, Liu J, Shi C, Wang S (2023). Comparative chloroplast genome analysis of Citrus (Rutaceae) species: insights into genomic characterization, phylogenetic relationships, and discrimination of subgenera. Sci Hort.

[2] Altaf N, Khan AR, Hussain J (2008). Fruit variability in Kinnow mandarin (*Citrus reticulata*). Pak J Bot.

[3] Pruvost OB, Boher C, Brocherieux M, Nicole, Chiroleu F (2002). Survival of *X. Axonopodis* Pv. *Citri* in leaf lesions under tropical environmental conditions and simulated splash dispersal of inoculum. Phytopathology.

[4] Sahi ST, Ghazanfar MU, Afzal M, Rashed A, Habib A (2007). Incidence of citrus canker disease caused by *Xanthomonas campestris* Pv. *Citri* (Hasse) dows on Kinnow (*Citrus reticulata*) and its chemotherapy. Pakistan J Bot.

[5] Gottwald TR, Hughes G, Graham JH, Sun X, Riley T (2001). The citrus canker epidemic in Florida: the scientific basis of regulatory eradication policy for an invasive species. Phytopathology.

[6] Shinde PB, Suryawanshi JS, Sethi BP, Deokar CD. In-vitro cultural studies and evaluation of different antibiotics and fungicides against Xanthomonas axonopodis pv. citri. causing citrus canker on Sai Sarbati variety of lime. 2023.

[7] Graham JH, Gottwald TR, Cubero J, Achor DS (2004). *Xanthomonas axonopodis* Pv. Citri: factors affecting successful eradication of citrus canker. Mol Plant Pathol.

[8] Shahbaz E, Ali M, Shafiq M, Atiq M, Hussain M, Balal RM, Shahid MA (2022). Citrus Canker Pathogen, its mechanism of infection, eradication, and impacts. Plants.

[9] Schubert TS, Sun X. Bacterial citrus canker. Plant Pathol J. 2003;Circular 377:1–6. Fl. Dep. Agric. and Cons. Services. Division. Plant industry.

[10] Das A (2003). Citrus canker. A review. J Appl Hortic.

[11] Adaskaveg JE, Schnabel G, Ritchie DF, Förster H. Common Preharvest Diseases of Peach and Nectarine Caused by Fungi and Bacteria: Biology, Epidemiology and Management. In *Peach* 2023; 261–342. GB: CABI.

[12] Machado-Moreira B, Richards K, Brennan F, Abram F, Burgess CM (2019). Microbial contamination of fresh produce: what, where, and how?. Compr Rev Food Sci Food Saf.

[13] Singh BK, Delgado-Baquerizo M, Egidi E, Guirado E, Leach JE, Liu H, Trivedi P. Climate change impacts on plant pathogens, food security and paths forward. Nat Rev Microbiol. 2023; 1–17. 10.1038/s41579-023-00900-7 PMC10153038 37131070

[14] Abbas T, Ahmad I, Nawaz R, Nazim M, Gatasheh MK, Alamri AM, Muneeb A. Physiological responses and antioxidant properties of Citrus reticulata under different abiotic stresses mitigated by endogenous melatonin. *Scientia Horticulturae*, 2023; *322*, p.112442.

[15] AOAC. Official Methods of Analysis. Association of Official Analytical Chemists. Inc., 15th Ed. Arlington, USA. 2006.

[16] Stall RE, Miller JW, Marco GM, de Echenique BC. Population dynamics of Xanthomonas citri causing cancrosis of citrus in Argentina. In *Proceedings of the Florida State Horticultural Society*. 1980. *93*, 10–14.

[17] Ali A, Zeshan MA, Iftikhar Y, Abid M, Ehsan SF, Ghani MU, Khan AA (2020). Role of plant extracts and salicylic acid for the management of Chili veinal mottle virus disease. Pakistan J Phytopathol.

[18] Pruvost O, Gottwald TR, Brocherieux C (1999). The effect of irrigation practices on the spatio-temporal increase of Asiatic citrus canker in simulated nursery plots in Reunion Island. Eur J Plant Pathol.

[19] Grecco ED, Silveira LFV, de Souza Lima VL, Pezzopane JEM (2014). Ecophysiological aspects of sun and shade leaves of Ponkan tangerine (*Citrus reticulata* Blanco). Idesia.

[20] Nawaz R, Abbasi NA, Hafiz IA, Khan MF, Khalid A (2021). Environmental variables influence the developmental stages of the citrus leaf miner, infestation level and mined leaves physiological response of Kinnow mandarin. Sci Rep.

[21] Ilyas A, Ashraf MY, Hussain M, Ashraf M, Ahmed R, Kamal A (2015). Effect of micronutrients (Zn, Cu and B) on photosynthetic and fruit yield attributes of citrus reticulata Blanco var. Kinnow. Pak J Bot.

[22] Budiarto R, Poerwanto R, Santosa E, Efendi D, Agusta A. Agronomical and physiological characters of kaffir lime (*citrus hystrix*) seedling under artificial shading and pruning. Emirates J Food Agric. 2019; 222–30.

[23] Oikeh EI, Oriakhi K, Omoregie ES (2013). Proximate analysis and phytochemical screening of *Citrus sinensis* fruit wastes. Bioscientist J.

[24] Aggett PJ, Erdman JW, Macdonald IA, Zeisel SH (2012). Iron. Present Knowledge in Nutrition.

[25] Hermansen K (2000). Diet, blood pressure and hypertension. Brazil J Nutr.

[26] Simpkins WA, Louie H, Wu M, Harrison M, Goldberg D (2000). Trace elements in Australian orange juice and other products. Food Chem.

[27] Ukana DA, Akpakpan AE, Enin GN (2012). Evaluation of Proximate compositions and Mineral elements in the Star Apple Peel, pulp and seed. J Basic Appl Sci Res.

[28] Imeh U, Khokhar S (2002). Distribution of conjugated and free phenols in fruits: antioxidant activity and cultivar variations. J Agric Food Chem.

[29] Dhiman A, Nanda A, Ahmad S (2013). Metal analysis in *Citrus sinensis* peel and *Psidium guajava* leaf. Toxicol Int.

[30] Alina AR, Babji AS, Affandi S (2009). Nutritional quality of palm fat substituted chicken nuggets. Nutr Food Sci.

